# n-3 Polyunsaturated fatty acids decrease mucosal/epidermal reactions and enhance antitumour effect of ionising radiation with inhibition of tumour angiogenesis

**DOI:** 10.1038/sj.bjc.6601136

**Published:** 2003-09-09

**Authors:** B Wen, E Deutsch, P Opolon, A Auperin, V Frascogna, E Connault, J Bourhis

**Affiliations:** 1 Laboratoire UPRES EA No. 27-10 Radiosensibilité des tumeurs et tissus sains, Institut Gustave-Roussy, 39, rue Camille Desmoulins, 94805 Villejuif Cédex, France; 2 Centre National de la Recherche Scientifique Unité Mixte de Recherche 8121 and Université Paris XI, Institut Gustave-Roussy, 39, rue Camille Desmoulins, 94805 Villejuif Cédex, France; 3 Service de biostatistique et d'épidémiologie, Institut Gustave-Roussy, 39, rue Camille Desmoulins, 94805 Villejuif Cédex, France

**Keywords:** n-3 polyunsaturated fatty acids, epidermal/mucosal reactions, antitumour effect, cyclooxygenase-2, ionising radiation

## Abstract

The purpose of this study was to evaluate the effect of n-3 polyunsaturated fatty acids (n-3 PUFAs) on normal tissue (lip mucosa) and tumour growth when combined with ionising radiation. The oral region (snout) of C57 black mice was irradiated with 16.5 Gy and n-3 PUFAs (100 *μ*l) were injected intravenously for 2 weeks. After exposure to irradiation, the degree and duration of the acute reactions decreased significantly when mice were treated with n-3 PUFAs as compared to the control group. Interestingly, the range of the reactions in the n-3 PUFAs-treated group compared favourably to the group receiving amifostine (27.5 mg/kg i.v.). the effect of n-3 PUFAs was further evaluated in HEP-2 human carcinoma xenograft transplanted in nude mice. An inhibition of tumour growth was observed when mice were treated with n-3 PUFAs alone and this effect was maximal when combined with irradiation. Similar results were obtained using eicosapentaenoic acid. The effect of n-3 PUFAs was associated with inhibition of angiogenesis and tumour proliferation, and significantly decreased expression of cyclooxygenase-2. In conclusion, n-3 PUFAs administration decrease mucosal response, while moderately enhancing the antitumour effect of irradiation. The magnitude of the differential effect suggests that n-3 PUFAs need to be further investigated in the clinic.

The n-3 polyunsaturated fatty acids (PUFAs), including eicosapentaenoic acid (EPA) and docosahexaenoic acid (DHA), which are present at high concentrations in some fish oils, have attracted interest because of their importance in normal development (Neuringer *et al*, 1988), and for their role as dietary supplements in the prevention and treatment of chronic cardiovascular disease (Simopoulos, 1991), the treatment of arthritic disorders (Kremer, 1991) and diabetes mellitus (Malasanos and Stacpoole, 1991). These agents have been evaluated in various clinical trials in which they have proved to be safe and well tolerated.

There is both epidemiologic and experimental evidence that n-3 PUFAs exert antitumour effects against some common cancers such as breast, colon, and prostate (Rose and Connolly, 1999). Multiple mechanisms are involved in this chemopreventive activity, including suppression of neoplastic transformation (Noguchi *et al*, 1997), cell cycle arrest (Albino *et al*, 2000), induction of apoptosis (Latham *et al*, 1999), antiangiogenesis and suppression of metastasis (Suzuki *et al*, 1997; Senzaki *et al*, 1998). However, a common feature of most biological effects is the inhibition of eicosanoid production from n-6 fatty acid precursors. n-3 PUFAs also exert an anticachectic effect in the animal model of hepatoma (Sauer *et al*, 1999; Smith *et al*, 1999).

Mechanistic experiments *in vitro* and *in vivo* support their potential use as cancer chemopreventive agents, and may support their use as auxiliary agents for cancer therapy. Since the interaction of n-3 PUFAs and ionising radiation has not been studied so far, the objective of the present study was to explore the effect of n-3 PUFAs on normal tissue and tumour response, when combined with ionising radiation.

## MATERIALS AND METHODS

### Reagents

The emulsion for perfusion, Omegaven®, (Fresenius, Kabi, France), containing 2% EPA and 2% DHA was used as a source of n-3 PUFAs. The 20% Intralipide® emulsion for perfusion (Fresenius, Kabi, France), which contains 20% purified soja oil, was used as a source of fatty acids devoid of n-3 PUFAs. Pure n-3 PUFAs, *cis*-5,8,11,14,17 EPA (Sigma Chemical Co., St Louis, MO, USA), 99% pure, were complexed to BSA according to the method described previously (Lai *et al*, 1996), aliquoted and stored at –20°C until use. Amifostine® was purchased from (Schering-Plough, Kenilworth, NJ, USA).

### Cell line

HEP-2, a human head and neck cancer squamous cell line, was maintained in RPMI 1640 medium (Sigma Chemical Co., St Louis, MO, USA), supplemented with 10% fetal calf serum, penicillin 100 *μ* ml^−1^ and streptomycin 100 *μ*g ml^−1^, 2 mM L-glutamine in a 5% carbon dioxide humidified incubator.

### Animals

Female C57 black mice and BALB/c nude mice, 6–8-week old, were purchased from Janvier CERT 53940 (Le Genest, St Isle, France). During the experimental period, the animals were housed five to seven per cage with sterilised white pine chip as bedding. The animal room was kept in a specific pathogen-free condition, and controlled for temperature (22±2°C), light (12-h) and humidity (60±10%). Animals used in this study were maintained in facilities in accordance with current regulations and observing ‘Principles and Guidelines for the Use of Animals in Research’ Issued by the French government according to the European community rules (Workman, 1998).

### Diets

The experimental basal diet used in this study was food (R.03 U.A.R., F91360, Villemosson sur Orge). Fresh diet was provided, and all remaining food was discarded, twice a week. Liquid food was used in every group during the evaluation of mucosal response. The liquid food (RENUTRYL® 500, Nestlé Clinical Nutrition, France) was substituted to the basal diet during the 3 weeks following irradiation.

### Lip epidermal/mucosal reactions to ionising radiation

The oral region (snout) of female C57 black mice was selectively irradiated with 225 kV X-rays (HVL 0.5 mm Cu, dose rate 0.7 Gy min^−1^ at 25.5 cm from the target, 250 kV Phillips). The mice were irradiated without anaesthetic using a specially constructed jig which held the mice tightly under the axillae, allowing to hold the snout centrally. Five to six mice were irradiated ventrally with a single dose of 16.5 Gy, with the whole body shielded by lead, except the anterior part of the snout. Dosimetry was carried out using a small Farmer–Baldwin ionisation chamber placed at the snout position within the mouse jig. At the same time, 100 *μ*l of Omegaven® was injected at the inner epicanthus with anaesthesia over a period of 2 weeks, 5 times a week. The same volume of Intralipide® or amifostine crystalline intraven (27.5 mg kg) was used as a control. At first, the mice were observed and weighted once every 2 days. After the appearance of reactions on the transitional zone and epidermal surface of the lips, they were routinely observed and the animals were weighted daily. Lip epidermal/mucosal reactions were scored using the Parkins scoring system giving a maximum score of 7 (Table 1

**Table 1:** Parkins' scoring system for lip reactions of mice

**Oedema score**	
0.5	50–50 doubtful if any swelling
1	Slight but definite swelling
2	Severe swelling
	
**Other scores**	
0.5	50–50 doubtful if abnormally pink
1	Slight but definite reddening
2	Severe reddening
3	Focal desquamation
4	Exudate or crusting involving about 1/2 lip area
5	Exudate or crusting involving more than 1/2 lip area

Separate scores for oedema (swelling) of lips, which is added to the other scores, giving a total of seven maximum scores.

) (Parkins *et al*, 1983; Maggiorella *et al*, 2001).

### Tumour irradiation procedure and response measurement

Three million cells in 0.1 ml of HEP-2 were implanted subcutaneously into the right flank of nude Balb/c mice and the animals were randomly allocated to control and treatment group when the size of tumour reached a mean of 5 mm, (*n*=5). Two sources of n-3 PUFAs were used for the study. We used a pure n-3 PUFA solution as a positive control (pure EPA combined with bovine serum albumin (BSA)) or the n-3 PUFAs alone or combined with irradiation, injections were performed during 2 weeks, 5 times per week. Irradiation consisted of a 15 Gy dose delivered in two 7.5 Gy fractions to the tumour with a Philips RT 250 radiation source operating at 225 kV X-rays with a 0.5 mm Cu filter. Irradiation was selectively delivered to the tumour by shielding the rest of the body with lead. Tumour response was assessed from relative change in the tumour volume in relation to the volume at the beginning of therapy. The tumour volume was calculated from the greatest transverse (width) and longitudinal (length) diameter of the tumour using the formula: tumour volume=length × width^2^/2 (Euhus *et al*, 1986). Measures were taken at the beginning of treatment, at the end of treatment and then twice weekly for 7 weeks. The mice were inspected daily during the period of experiment for any signs of disease or distress. At the end of the observation period, mice were killed and tumours were removed and processed for histopathological evaluation.

For tumour control probability evaluation, tumours were locally irradiated at various doses in two fractions; pure n-3 PUFA solution injections were performed during 2 weeks, 5 times per week. Radiation doses yielding 50% tumour control at 90-day values were computed by the logit method of analysis (Finnley, 1952).

### Assessment of tumour vascularisation

To analyse the effect of n-3 PUFAs in HEP-2 tumour *in vivo*, we performed histological examination of tumour samples. Tumours were collected on day 5 after the first injection of n-3 PUFAs and/or irradiation. Immunohistochemistry was used to assess the intratumoural vascularisation within the different experimental groups. Tumour tissues were fixed in 100% ethanol embedded in paraffin and 5-*μ*m sections were prepared and routinely stained with haematoxylin–eosin–saffranin. Endogenous peroxidase activity was quenched by 3% H_2_O_2_ for 5 min.

The sections were placed in coverplates (Shandon, Life Sciences Technology, Cergy-Pontoise, France) and incubated with blocking serum Optimax wash buffer 1 : 10 (BioGenex, San Ramon, CA, USA) 10 min. This step was followed by incubation with a mixture of two rat primary antibodies raised against mouse platelet endothelial cell adhesion molecule (Pecam1, PharMingen, Becton-Dickinson, France, #Mec13.3 and 390 clones) at a dilution of 1 : 50 for 1 h. Slides were then incubated with a goat anti-rat biotin-conjugated antibody 1 : 50 (PharMingen) and subsequently with streptavidin-peroxidase (Dako, Trappes, France) 1 : 100 for 30 min and with the DAB Powervision kit (Immunovision Technologies Microm, France) for 10 min, counterstained with Mayer's haematoxylin and mounted. Evaluation of tumour vascularisation was achieved by selecting representative for semiquantitative assessment of vascularisation, excluding necrotic and fibrotic areas.

Highly vascularised tumours exhibit microvessels within the peripheral non-necrotic tumour area associated with a marked fibrotic peritumoural vascular zone.

Tumours of intermediate level of vascularisation were associated with an absence of vessels in the tumour itself, but with a persistence of a well-vascularised fibrotic capsule.

Tumours of low level of vascularisation are associated with an absence of microvessels both within the tumour and at its periphery.

### Analysis of COX-2 expression by Western blot

HEP-2 cells were treated with 100 nM ml^−1^ EPA and/or 5 Gy ionising irradiation for 48 h. After rinsing with cold PBS, cells were lysed with Tween-20 lysis buffer (50 mM HEPES pH 7.4, 150 mM NaCl, 1 mM EDTA, 1% NP-40, 1 mM DTT, 50 mM NaF, 1 mM PMSF, 0.5 mM Na_3_VO_4_), proteinases inhibitor and sonicated. Equal amounts of proteins were analysed by 10% Sodium dodecyl sulphate–polyacrylamide electrophoresis (SDS–PAGE). Thereafter, proteins were transferred on to nitrocellulose membranes and analysed using rabbit anti-COX-2, mouse *β*-actin. Proteins were detected via incubation with horseradish peroxidase-conjugated secondary antibodies and the enhanced chemiluminescence detection system. *β*-Actin was used as a control for loading.

### Reverse transcriptase–polymerase chain reaction for COX-2

RNA isolation from HEP-2 was performed according to the manufacturer's protocol (Promega, Charbonieres, France). Aliquots from RNA were used for the detection of COX-2 and the control *β*2 microglobulin mRNA (*β*2*μ*). Total RNA was reverse-transcribed with 5 u of AMV reverse transcriptase for 45 min at 48°C. (PCR) reaction (94°C for 30 s, 60°C for 1 min, 68°C for 2 min and an extension 72°C for 10 min) was carried out with 100 *μ*M primers and 5 U of Taq polymerase in 50 *μ*l. The COX-2 primer pairs were 5′-CGAGGTGTATGTATGAGTGTG-3′, and 5′-TCTAGCCAGAGTTTCACCGTA-3′ generating a 550 bp product (Subbarayan *et al*, 2001). The *β*2 microglobulin primer pairs consisted of 5′-CATTCGGGCCGAGATGTC-3′ and 5′-CTCCAGGCCAGAAAGAGAGAGTAG-3′. A volume of 15 *μ*l of the Reverse transcriptase–polymerase chain reaction for COX-2 (RT–PCR) products was separated on a 1.5% agarose gel and visualised under ultraviolet light.

### Statistical analyses

Every 2 days, erythema-mucositis scores from day 6 to day 32 were studied by a mixed model. Among mice treated by reverse transcriptase (RT) alone and among mice treated by RT+omegaven, the scores were significantly different between the two experiments. Thus, the effect of omegaven was tested by a mixed model comparing scores between mice treated by RT alone and mice treated by RT+omegaven and taking into account the experience effect.

Two other end points were studied: the duration between the first day and the last day with a score equal or higher than 2 and the maximum score reached during the follow-up period. The comparison of these two criteria between the RT alone and RT+omegaven was performed using the Wilcoxon nonparametric rank test within each experience. These two criteria were also compared between RT+amifostine and RT+omegaven groups.

## RESULTS

### Effect of n-3 PUFAs on lip epidermal/mucosal reactions to X-irradiation

The mouse lip epidermal/mucosal reactions after irradiation with 16.5 Gy began at 8 days, reached a peak between 12 and 16 days and then returned to low values. The mixed model that studied every 2 days erythema-mucositis scores from days 6 to 32 and taking into account the experience effect showed that scores were significantly lower in the RT+omega group than in the RT alone group (*P*=0.01). The degree and duration of these acute reactions decreased significantly when mice were treated with n-3 PUFAs (Tables 2

**Table 2:** Description of the Wilcoxon nonparametric rank test

	**Duration of score ⩾2 (days)**	**Maximum score**
	**Median (range)**	**Median (range)**
RT experiment 1 (*n*=5)	14 (11–16)	5.5 (4.5–6)
RT experiment 2 (*n*=5)	8 (7–9)	6 (5–7)
RT+intralipid experiment 1 (*n*=5)	11 (8–15)	5 (5–6)
RT+amifostine experiment 2 (*n*=4)	9 (8–10)	3.5 (3–4)
RT+omegaven experiment 1 (*n*=5)	6 (4–8)	2.5 (2.5–3.5)
RT+omegaven experiment 2 (*n*=6)	7 (5–8)	4 (4–5)

RT=reverse transcriptase.

and 3

**Table 3:** P-value of Wilcoxon non parametric rank test

	**Duration of score ⩾2**	**Maximum score**
Rt *vs* RT+omegaven		
Experiment 1	0.03	0.03
Experiment 2	0.18	0.03
RT+amifostine *vs* RT+omegaven (experiment 2)	0.054	0.10
RT *vs* RT+intralipid (experience 1)	0.24	0.92
RT *vs* RT+ amifostine (experience 2)	0.27	0.046

**Figure 1:**
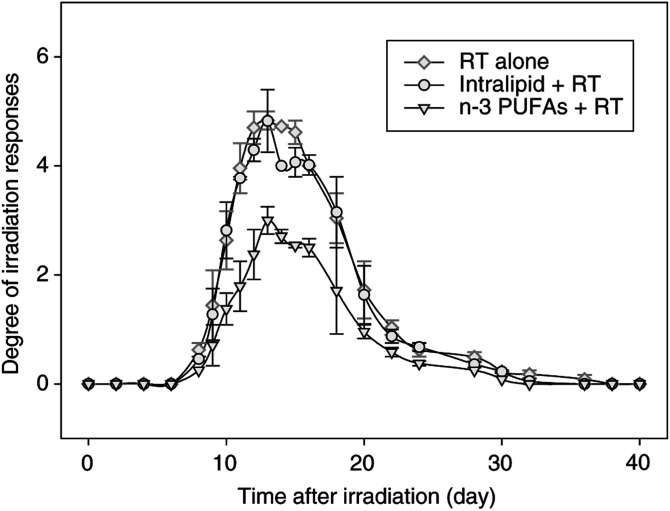
Epidermal/mucosal lip reaction scores in mice, which were the mean of two experiments for irradiation alone (16.5 Gy) and for irradiation plus Intralipid (soja oil) and irradiation plus n-3 PUFAs.

**Figure 2:**
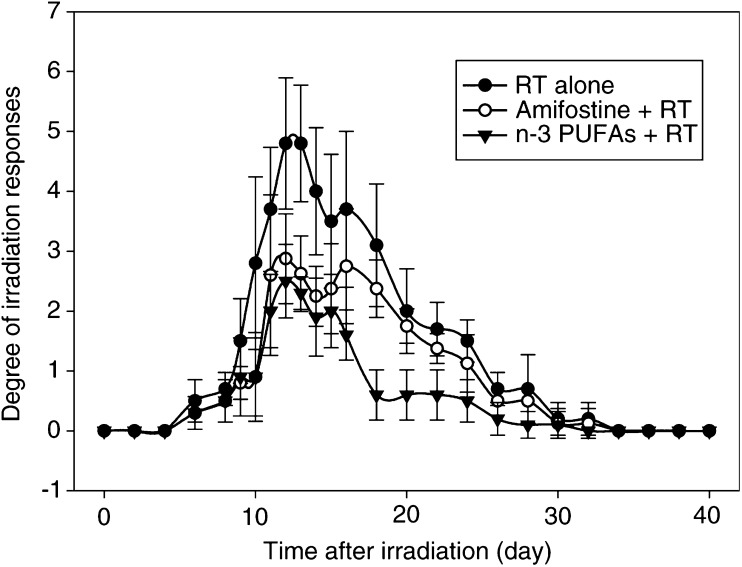
Epidermal/mucosal lip reaction scores in mice; *P*=0.0002 for irradiation alone and irradiation plus amifostine; *P*<0.0001 for irradiation alone and irradiation plus n-3 PUFAs.

RT=reverse transcriptase.

). No effect was seen when mice were treated with Intralipide® Emulsion containing 20% soja oil (Figure 1

). The degree of epidermal/mucosal reactions decreased when mice were treated with amifostine before irradiation. Figure 2

shows a comparison between the effect of amifostine and n-3 PUFAs. The radioprotective effect was slightly more pronounced in the n-3 PUFAs group than in the amifostine group, although not statistically different (Table 3).

### Effects on tumour growth

**Figure 3:**
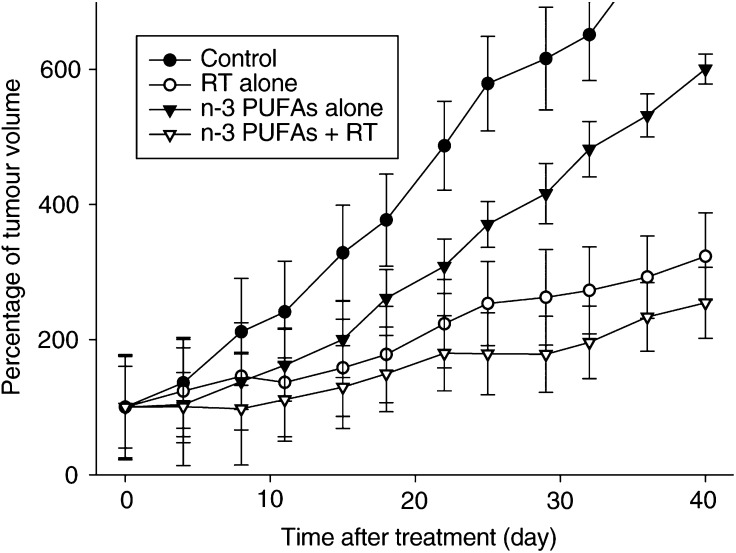
Change in tumour volume of tumour xenografts (HEP-2) in nude mice irradiated (RT) to a total dose of 15 Gy in two fractions of 7.5 Gy. Values at each point are the mean ratios of the observed tumour volume at a considered time divided by the initial tumour volume. Error bars represent the mean±the standard error of the ratio.

**Figure 4:**
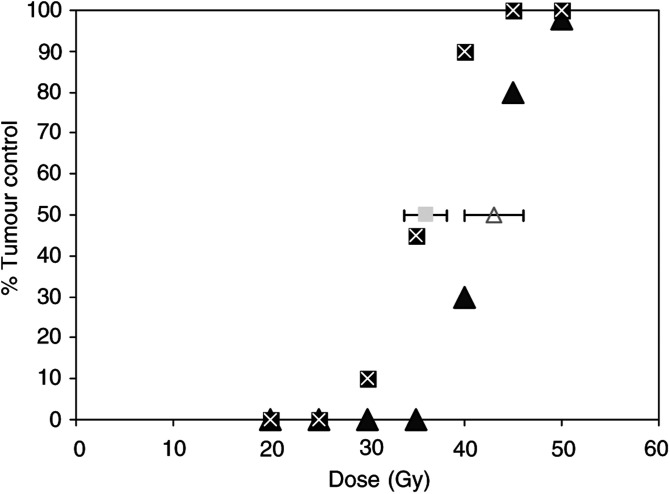
Tumour control probability at 90 days for 8 mm diameter HEP-2 tumours according to the radiation dose for animals treated with irradiation alone (dark triangles), or irradiation and n-3 PUFA (dark squares). Clear triangles and clear square, respectively, represent the dose curing 50% of the animals for irradiation alone and irradiation plus n-3 PUFA; error bars show the 95% confidence interval.

As shown in Figure 3

, a moderate tumour growth inhibition was observed in mice receiving an injection of n-3 PUFAs or irradiation alone, compared to the control group. The growth rate of the tumour in the combined group was lower than in the n-3 PUFAs alone group or irradiation alone group. A comparable inhibitor effect on tumour growth was observed when mice were treated with pure EPA (data not shown). However, the differences in tumour growth retardation did not reach significance (*P*=0.14 using Wilcoxon's nonparametric rank test). No significant toxicity or side effects were observed in the various treatment groups (data not shown) (Figure 4

).

**Figure 5:**
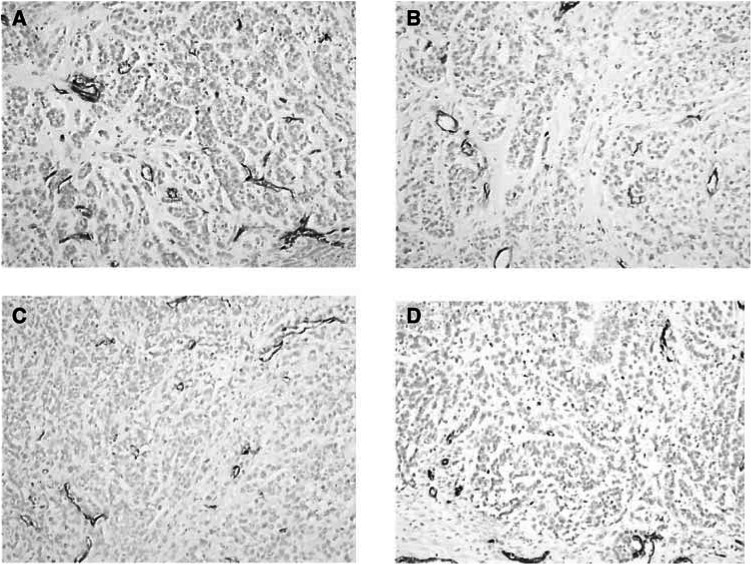
Detection of vessels within the tumour of HEP-2 tumour xenograft: Pecam-1 endothelial immunostaining, magnification × 100. (**A**) Control group, highly vascularised tumour with thick and large vascular channels. Presence of large vessels located at the periphery of the tumour. (**B**) Irradiation alone, moderately vascularised tumours. (**C**) n-3 PUFA alone, moderately vascularised tumours. (**D**) Irradiation combined with n-3 PUFA, marked inhibition in tumour vascularisation.

**Figure 6:**
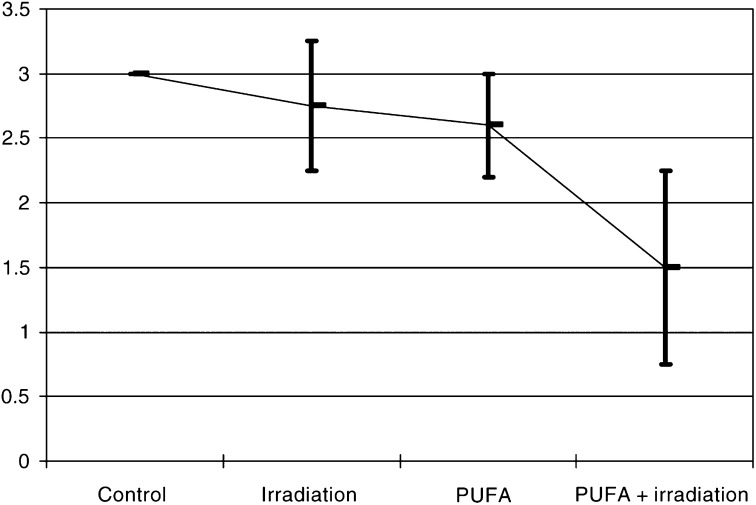
Evaluation of the degree of tumour vascularisation in HEP-2 tumour xenograft according to the scoring of HEP-2 tumours from 20 animals that were either untreated, treated by radiation alone, n-3 PUFA alone or both. The diagram shows the mean scoring for each group, error bars represent the standard error. Differences between the irradiation group and irradiation plus n-3 PUFA were statistically significant, *P*<0.05.

The histological examination of the tumours showed a typical viable squamous cell carcinoma with various degrees of necrosis in the control group, fewer focal areas of viable cells surrounded by fibrocytes in the n-3 PUFAs group and almost undetectable tumour cells in the group of n-3 PUFAs plus irradiation (Figure 5

). Immunohistochemical staining with mouse anti-CD31 antibody indicated that n-3 PUFAs significantly inhibit the tumour, whcih was highly vascularised with thick and large vascular channels in the control group, moderately vascularised in the irradiation alone and the n-3 PUFAs groups. The formation of angiogenesis was markedly inhibited in the n-3 PUFAs combined with the irradiation group (Figure 6

).

### Decreased expression of COX-2

**Figure 7:**
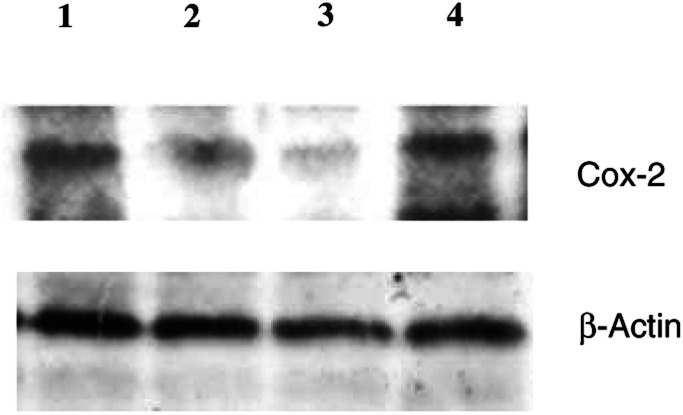
Representative Western blot expression of COX-2 and protein in HEP-2 cells were treated with EPA (pure n-3 PUFA) 100 *μ*g ml^−1^ and/or irradiated with 5 Gy. The interval for combined treatment was 24 h. All cells were harvested at 48 h treatment. Lane 1: control, lane 2: n-3 PUFAs 100 *μ*g ml^−1^, lane 3: irradiation with 5 Gy plus n-3 PUFAs 100 *μ*g ml^−1^, lane 4: 5 Gy irradiation.

**Figure 8:**
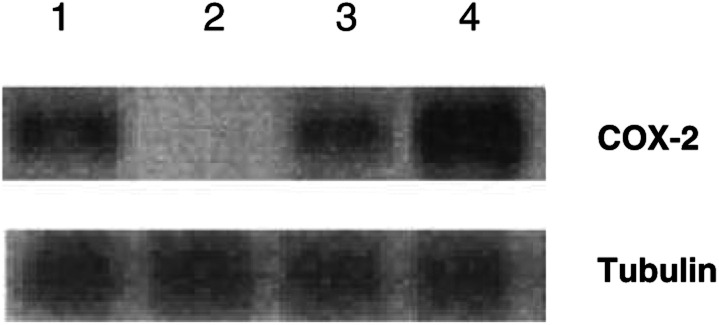
Expression of COX-2 mRNA in HEP-2. HEP-2 cells were treated with EPA 100 *μ*g ml^−1^ and/or irradiated with 5 Gy. The interval for combined treatment was 24 h. All cells were harvested at 48 h after treatment. Lane 1: control, lane 2: n-3 PUFAs 100 *μ*g ml^−1^, lane 3: n-3 PUFAs 100 *μ*g ml^−1^+5 gy, lane 4: 5 Gy irradiation alone.

Several potential molecular markers were studied in HEP-2 cells, including COX-2 expression for which significant changes were observed. Cells were treated with EPA 100 *μ*g ml^−1^ and/or 5 Gy irradiation for 48 h. COX-2 protein was upregulated by irradiation in a time-responsive manner, but it was downregulated by EPA in a concentration-responsive and time-responsive manner (data not shown). Figure 7

illustrates the expression of COX-2 proteins, as determined by Western immunoblot analysis in HEP-2 cells. The downregulation of COX-2 was more pronounced when combined with irradiation. Further study using RT–PCR showed that EPA was able to inhibit COX-2 expression at the transcriptional level (Figure 8

).

## DISCUSSION

Both epidemiologic and experimental studies have suggested that n-3 PUFAs can exert a chemopreventive effect against some common tumours and multiple mechanisms have been involved in this activity, including suppression of neoplastic transformation (Noguchi *et al*, 1997), cell cycle arrest (Albino *et al*, 2000), induction of apoptosis (Latham *et al*, 1999), antiangiogenesis and suppression of metastasis (Ligo *et al*, 1997; Senzaki *et al*, 1998). This study was designed to assess the possible interaction of n-3 PUFAs and ionising radiation, which has not been reported before.

The n-3 PUFAs were found to decrease the mucosal/epidermal response of irradiation (Figures 1 and 2). The mechanisms involved in the protection of the mucosa from irradiation are not known. Interestingly, the effect was of the same magnitude as that observed with amifostine, which is so far one of the most efficient radioprotective agents. It has been reported that the n-3 PUFAs are immunomodulatory and have been shown to suppress endotoxin-induced production of proinflammatory cytokines such as IL-1 and tumour necrosis factor (TNF) by peripheral blood mononuclear cells (PBMC) from healthy volunteers (Meydani *et al*, 1993). Studies of weight-losing pancreatic cancer patients receiving high-purity EPA have demonstrated suppression of PBMC IL-6 production (Wigmore *et al*, 1997). Serhan *et al* (2000) have reported that n-3 PUFA exerts antimicroinflammatory lipid signals via COX-2, which might be involved in its radioprotective action.

Owing to the marked radioprotective effect on normal tissue, it was essential to determine whether these agents could also exert a tumour protection after irradiation. Interestingly, no tumour protection was observed in HEP-2 squamous cell carcinoma tumour, whereas a moderate antiproliferative effect of n-3 PUFAs was found with the suggestion that the combination of n-3 PUFAs and ionising radiation led to a more important growth delay than ionising radiation alone. This effect might be mediated by several modifications induced by n-3 PUFAs including COX-2 inhibition, downregulation of the antiapoptotic bcl-2 gene and finally by an antiangiogenic effect (Figures 5 and 6).

In contradiction with previous reports, we did not find a significant antiangiogenic effect of n-3 PUFAs when given alone; this might be related to the relatively short period of overall n-3 PUFAs administration in our study.

The observed n-3 PUFAs induced downregulation of mRNA transcriptional level and protein COX-2, which is in good agreement with previous reports that a short-term dietary intervention in men with prostate cancer led to a significant increase in omega-3/omega-6 fatty acid ratios in adipose tissue, and the potential for the diet to prevent the development and progression of prostate cancer by way of altered expression of COX-2 expression in prostatic tissue specimen which was determined by semiquantitative RT–PCR (Aronson *et al*, 2001). In addition, modulation of n-3 PUFAs production via the influence on COX-2 activity and the suppression of apoptosis may also play a key role in high-fat mixed lipids diet-induced rat colon tumorigenesis (Rao *et al*, 2001). Our study is in agreement with these data and is the first to show that n-3 PUFAs were able to enhance the antitumour effect of radiation therapy partly by decreasing the expression of COX-2 induced by irradiation.

In conclusion, our findings indicated that n-3 PUFAs decrease mucosal/epidermal reactions of ionising radiation with the same magnitude as that of a reference radioprotective agent, amifostine, while enhancing the antitumour effect of radiation therapy. The antitumour effect was associated with the inhibition of angiogenesis and tumour proliferation, and a decreased expression of COX-2. The results of this study strongly suggest that the combination of n-3 PUFAs and ionising radiation should be further tested in patients.
